# Clinical Significance of Clonal Hematopoiesis of Indeterminate Potential in Hematology and Cardiovascular Disease

**DOI:** 10.3390/diagnostics12071613

**Published:** 2022-07-02

**Authors:** Gregor Hoermann

**Affiliations:** MLL Munich Leukemia Laboratory, 81377 Munich, Germany; gregor.hoermann@mll.com; Tel.: +49-89-99017-315

**Keywords:** clonal hematopoiesis of indeterminate potential, clonal cytopenia of unknown significance, myeloid neoplasms, cardiovascular risk, cell-free DNA

## Abstract

Liquid profiling uses circulating tumor DNA (ctDNA) for minimal invasive tumor mutational profiling from peripheral blood. The presence of somatic mutations in peripheral blood cells without further evidence of a hematologic neoplasm defines clonal hematopoiesis of indeterminate potential (CHIP). CHIP-mutations can be found in the cell-free DNA (cfDNA) of plasma, are a potential cause of false positive results in liquid profiling, and thus limit its usage in screening settings. Various strategies are in place to mitigate the effect of CHIP on the performance of ctDNA assays, but the detection of CHIP also represents a clinically significant incidental finding. The sequelae of CHIP comprise the risk of progression to a hematologic neoplasm including therapy-related myeloid neoplasms. While the hematological risk increases with the co-occurrence of unexplained blood count abnormalities, a number of non-hematologic diseases have independently been associated with CHIP. In particular, CHIP represents a major risk factor for cardiovascular disease such as atherosclerosis or heart failure. The management of CHIP requires an interdisciplinary setting and represents a new topic in the field of cardio-oncology. In the future, the information on CHIP may be taken into account for personalized therapy of cancer patients.

## 1. Introduction

Liquid profiling is an emerging technology that relies on the analysis of circulating tumor DNA (ctDNA) for non-invasive tumor mutational profiling [1]. The usage of peripheral blood instead of tumor biopsies led to the term liquid biopsy and enables sequential assessment of tumor mutations for early detection, therapy response monitoring, and therapeutic management of cancer patients. While circulating tumor cells (CTC), exosomes, and tumor-derived platelets have also been applied to study tumor characteristics in the peripheral blood, the molecular analysis of ctDNA is best established for liquid profiling of cancer. PCR-based techniques and next generation sequencing (NGS) are used to detect and quantify somatic mutations in ctDNA [2]. In many cases, ctDNA is only a small fraction of the total cell-free DNA (cfDNA) in the plasma and may vary from as low as 0.01% up to a large fraction of cfDNA, depending on the tumor type, the tumor mass (often reflected by the tumor stage) and the type of treatment [3]. The remaining predominant fraction of cfDNA originates from hematopoietic cells [4]. In the case of a hematologic neoplasm, somatic mutations derived from clonal hematopoietic cells can also be detected in cfDNA. While the concurrence of an overt hematologic neoplasm with a solid cancer is rather rare, the presence of clonal hematopoiesis of indeterminate potential (CHIP) is a frequent finding in cancer patients [5].

In the year 2014, three large studies detected a high prevalence of putative somatic mutations in the peripheral blood of healthy individual using exome sequencing data [6,7,8]. The mutational spectrum showed a large overlap to that of myeloid neoplasms, in particular myelodysplastic syndromes (MDS), and including typical hematopoietic driver mutations [9]. This condition was first termed age-related clonal hematopoiesis (ARCH) as its prevalence was clearly age dependent [7]. While the prevalence of ARCH was negligible in young individuals (<40 years), it steadily increased between the age of 40 and 60, and >10% of apparently hematologically healthy individuals harbored large hematopoietic clones by the age of 70 [6,7,8,10]. Now this condition is typically referred to as CHIP which is defined by the detection of somatic mutation that is commonly associated with clonal expansion of hematopoietic cells whereas the criteria for the diagnoses of any hematological neoplasms are not met [11]. The definition also includes a minimal variant allele fraction (VAF) of 2% in the peripheral blood (corresponding to ~4% of leukocytes harboring a heterozygous mutation) to discriminate CHIP from nearly ubiquitous extremely small hematopoietic clones [11]. CHIP will also be included as an entity in the 5th edition of the World Health Organization Classification of Hematolymphoid Tumors where it will be defined similarly by the presence of somatic mutations of myeloid malignancy-associated genes detected in the blood or bone marrow with a VAF ≥ 2% (≥4% for X-linked gene mutations in males) in individuals without a diagnosed hematologic disorder or unexplained cytopenia [12].

Table 1 lists the genes typically mutated in individuals with CHIP [12]. While some of the CHIP mutations are specific for hematopoietic clones and neoplasms, others are also frequently found in solid cancers. As these CHIP-derived somatic mutations are also present in a substantial proportion of cfDNA, they represent a relevant source of biological noise in liquid profiling [13]. In fact, CHIP-associated mutations are frequently detected by liquid biopsy. The relevance of the biological noise caused by CHIP increases with the size and the nature of the target region in liquid profiling. While the analysis of *EGFR* hotspot mutations is typically not affected, as *EGFR* is not recurrently mutated in CHIP, larger gene panels including common cancer genes (e.g., *TP53*) are prone to false positive results as they may detect mutations rather derived from CHIP than from ctDNA of a solid cancer [14,15]. While follow-up analysis of known patient-specific cancer mutations in ctDNA may potentially overcome this limitation, the clinical relevance is highest in a screening setting where the tumor is not known before. Thus, CHIP is one of the issues that hamper the usage of liquid profiling for early stage cancer detection. A number of techniques are therefore applied to reduce the CHIP-associated error rate and to discriminate tumor-associated from CHIP-associated mutations. The plethora of approaches include paired genotyping of peripheral blood cells together with cfDNA [16,17,18], size exclusion relying on differences in the size of cfDNA molecules from tumor cells compared to leukocytes [19,20], or mutational signature analysis [21].

While CHIP represents a technical challenge for the interpretations of results from NGS-based liquid profiling, the incidental detection of CHIP itself may be of clinical significance [23]. This review summarizes the clinical relevance of CHIP in hematologic as well as non-hematologic diseases with a special focus on the cardiovascular risk.

## 2. CHIP and Hematologic Neoplasms

CHIP is a risk factor for development of hematologic neoplasms. While the relative risk of CHIP carriers is significantly increased, the absolute risk of progression to a—mostly myeloid—hematologic neoplasm is approximately 0.5% to 1% per year [11]. This is very similar to the well-known risk for the progression from monoclonal B-cell lymphocytosis (MBL) to chronic lymphocytic leukemia (CLL), or from monoclonal gammopathy of undetermined significance (MGUS) to plasma cell myeloma [24]. However, the combination of CHIP with blood count abnormalities and/or specific mutational patterns substantially increases the risk of progression as recently reviewed in detail [25]. In the context of patients suffering from cancer, CHIP increases the risk for development of a therapy related myeloid neoplasm as sequelae of chemotherapy or radiation [26].

In the general population, the risk of progression to a hematologic neoplasm is moderate for CHIP carriers. Overall, the development of a hematologic neoplasm is roughly 10-times higher in individuals with CHIP [6,7]. Both myeloid and lymphoid neoplasms can be the sequelae of CHIP although myeloid neoplasms are by far more frequent [27]. The genetic pattern allows a discrimination of myeloid-like CHIP (characterized by mutations in the typical CHIP genes *DNMT3A*, *TET2*, and *ASXL1*) and lymphoid-like CHIP. Multiple mutations and in particular the combination of single and small nucleotide variants (SNVs) with rare mosaic chromosomal alterations (copy number variants, CNVs; or copy-neutral loss of heterozygosity, LOH) significantly amplify the risk of progression from hazard ratios < 10 to approximately 100 [27]. While CHIP in general is a premalignant condition with a moderate risk of progression, the term clonal hematopoiesis of oncogenic potential (CHOP) has been suggested for the presence of distinct driver mutations associated with a higher risk of progression [28,29]. However, the highest clinical relevance is associated with the combination of CHIP and unexplained blood count abnormalities not sufficient to meet the diagnostic criteria of a hematologic neoplasm.

CHIP carriers typically show a normal blood count and differential. Only an increase of red cell distribution width (RDW) is consistently associated with CHIP [7]. In some individuals, CHIP is accompanied by unexplained cytopenia (anemia, neutropenia, and/or thrombocytopenia) which is defined as clonal cytopenia of unknown significance (CCUS) [11,12,28]. Per definition, CCUS patients do not meet the diagnostic criteria of a hematologic neoplasm. Myelodysplastic syndromes (MDS) are hematopoietic stem cell neoplasms characterized by the presence of cytopenia and dysplasia [28]. In the vast majority of MDS patients, the clonal nature of the disease can be demonstrated by chromosomal analysis and molecular genetics that identify somatic genetic alterations in >90% of patients [30,31]. Thus, the differential diagnosis between CCUS and MDS primarily relies on the presence or absence of dysplasia in bone marrow morphology (Table 2). Patients who do not meet the diagnostic criteria of MDS but are diagnosed with CCUS are at a substantial risk for progression [32,33]. For specific mutational patterns involving mutations in splicing factor genes (e.g., *SF3B1*, *SRSF2*, or *U2AF1*) and/or multiple mutations, the progression risk of CCUS is ~20% per year [32]. Further studies confirmed the utility of specific mutational patterns and clonal metrics to define high-risk CCUS [34]. In contrast, the risk of progression is moderate if only a single mutation in the epigenetic modifier *DNMT3A* is observed [34]. In a study on persons aged ≥ 80 years, the clinical course of patients with high-risk CCUS was indistinguishable from that of overt myeloid neoplasms [35]. In summary, the consideration of mutational patterns and clonal metrics allow to predict the individual risk in patients with unexplained cytopenia. Molecularly defined high-risk CCUS and low risk MDS represent rather a continuous spectrum of disease development than two completely distinct diseases [25]. From a practical point of view, the presence of unexplained anemia, neutropenia, and/or thrombocytopenia is a red flag in an individual with CHIP as it represents at least CCUS but might also be a sign of an underlying occult hematologic neoplasm. While a closer hematologic monitoring is mandatory in this situation, a bone marrow examination is often required for the exclusion of an underlying MDS [28].

Unexplained cytopenia is not the only condition associated with an increased clinical significance of CHIP. Likewise, unexplained and persistent polycythemia, thrombocythemia, and/or leukocytosis should lead to a hematologic workup when CHIP is detected (Table 2). The clonal increase of mature blood cells is a hallmark of myeloproliferative neoplasms (MPN), such as essential thrombocythemia (ET), polycythemia vera (PV), primary myelofibrosis (PMF), chronic neutrophilic leukemia (CNL), or chronic eosinophilic leukemia (CEL). Contradictorily, anemia is common in patients with PMF due to the bone marrow fibrosis. The classical *BCR*–*ABL1* negative MPN ET, PV, and PMF are characterized by driver mutations in the genes *JAK2*, *MPL*, or *CALR* that lead to a ligand independent activation of JAK/STAT signaling [36]. The hotspot mutation *JAK2* V617F is the most prevalent genetic aberration found in more than 60% of patients with MPN; it is also observed in individuals with CHIP [6,7,8,37]. The presence of a MPN driver mutation together with a persistent increase in the blood count is a red flag that warrants a thorough hematologic workup in an individual with CHIP. On the one hand, it has been shown that MPN driver mutations are associated with respective blood count alterations in the general population even if the diagnostic criteria for MPN are not met. In line with this, MPN-associated bone marrow changes have been found in some of these cases suggesting that MPN might be underdiagnosed by current criteria and guidelines [38,39]. On the other hand, phylogenetic studies of clonal development have recently shown that the acquisition of *JAK2* V617F precedes the phenotypic diagnosis of MPN by 30 years on average with very slow expansion of the mutated clone over decades [40]. While these findings argue for a more differentiated consideration of *JAK2* V617F as diagnostic criterion for MPN (e.g., via a minimal required VAF), the cardiovascular risk—which is also a hallmark of *JAK2*-mutated MPN—is substantial in *JAK2*-mutated patients even if the diagnostic criteria for MPN are not met [41,42]. The diagnostic significance of CHIP-mutations in the context of polycythemia, thrombocythemia, and/or leukocytosis is not limited to the classical hotspot mutations in *JAK2*, *MPL*, or *CALR* as mutations in other genes are recurrently found in so called triple-negative MPN [43]. Likewise, the detection of clonal hematopoiesis is of clear diagnostic significance in patients with unexplained monocytosis and may lead to the diagnosis of chronic myelomonocytic leukemia (CMML) [44]. Subsequently, the term “clonal monocytosis of clinical significance” has been suggested for patients not meeting the morphologic criteria of CMML [44,45]. Finally, CHIP-mutations can also be accompanied by unexplained eosinophilia and are of diagnostic relevance in patients with CEL [46].

In the context of liquid profiling of cancer, it is of utmost importance that CHIP increases the risk of treatment-related myeloid neoplasms (tMN) in patients with solid cancer [47]. CHIP is highly prevalent in patients having undergone chemotherapy and/or radiation [5,48]. In particular, mutations in DNA damage response genes (*TP53*, *PPM1D*, *CHEK2*) are frequently observed, and were found to outcompete other clones when exposed to further cytotoxic therapies [49]. *PPM1D* mutations are most characteristic for tMN [50]. Further chemotherapy and/or radiation is a risk factor for the progression from CHIP to tMN [49]. The highest risk was observed for mutations in *TP53* or spliceosome genes (*SRSF2*, *U2AF1, SF3B1*). Furthermore, it has been suggested that the risk of tMN development could exceed the benefit of adjuvant chemotherapy in patients with high-risk CHIP and early-stage breast cancer [49]. While the expansion or evolution of the CHIP clone is frequently observed in patients undergoing chemotherapy and/or radiation, it was not found in patients undergoing immunotherapy with checkpoint inhibitors [51]. In summary, the evidence on the clinical significance of CHIP for tMN development in cancer patients is currently not sufficient to justify a modification of treatment guidelines [52]. Still, this association provides a rationale for future studies focusing of risk-adapted treatment decisions that take the presence of CHIP into account [49].

To summarize the hematologic risk accompanying CHIP, the least risk of progression is observed in the majority of CHIP carriers with small *DNMT3A* mutated clones showing neither additional mutations nor blood count abnormalities. Intensified hematologic monitoring has been suggested for CHIP clones with a larger VAF (>10%), mutations in high-risk genes—i.e., splicing factor genes (e.g., *SF3B1*, *SRSF2*, *U2AF1*), *TP53*, *PPM1D*, *RUNX1*, *IDH1*, *IDH2*, MPN-driver genes (*JAK2*, *MPL*, *CALR*)—additional mosaic chromosomal alterations, or mutations in multiple genes [53]. The combination of CHIP clones with unexplained blood count alterations warrants the respective hematologic workup to exclude an underlying occult hematologic neoplasm [25]. In cancer patients, the presence of CHIP has been associated with reduced survival. While this can be partly explained by the risk of tMN an additional role of proinflammatory tumor-associated macrophages on tumor progression in the context of CHIP has been suggested [49,54,55]. Further mechanistic and clinical studies are needed to decipher the complete picture of CHIP in solid cancer and to personalized tumor treatment taking the information on CHIP into account.

## 3. CHIP and Cardiovascular Disease

CHIP has not only been associated with the development of hematologic neoplasms but also with various non-malignant diseases including atherosclerotic cardiovascular disease, ischemic heart failure, chronic obstructive pulmonary disease, and autoimmune disorders [56]. While the pathophysiologic link between CHIP and myeloid neoplasms has long been anticipated, the connection between CHIP and cardiovascular disease is less obvious. The first studies identifying ARCH/CHIP in apparently healthy individuals already reported an association with cardiovascular endpoints as reviewed previously [10]. Jaiswal et al. described an increase in all-cause mortality that was rather linked to an increased risk of incident coronary heart disease and ischemic stroke but to the effect of the increased risk of hematologic neoplasms in the total cohort [7]. A subsequent landmark study analyzed four large case-control studies (in total 4726 participants with coronary heart disease and 3529 controls) and clearly established the association between CHIP and coronary heart disease with a 1.9 times greater risk for CHIP carriers after adjustment for the traditional cardiovascular risk factors age, sex, type 2 diabetes, and smoking [42]. Of note, while CHIP is rarely detected in younger individuals, it was substantially associated with for early-onset myocardial infarction (age < 50 years) with a hazard ratio of 4.0 [42]. This and other studies established CHIP as a new cardiovascular risk which is at least in a similar order of magnitude as traditional risk factors such as hyperlipidemia or smoking [24]. With regard, to individual genes, detailed analysis indicated a particularly high risk for *JAK2* (12.1-fold risk increase) compared to *DNMT3A*, *TET2*, and *ASXL1* (1.7 to 2.0 fold risk increase). Furthermore, a relatively large clone size with a VAF > 10% was needed to impose the cardiovascular risk [42]. The distinct effect of *JAK2* V617F on the cardiovascular risk was confirmed in a Japanese cohort studying MPN driver mutations [41]. In line, the presence of CHIP in individuals with unexplained erythrocytosis but not meeting the diagnostic criteria of MPN has been associated with an increased cardiovascular morbidity and mortality [57]. Finally, a recent meta-analysis including 32 studies with 56 cohorts that examined the association between CHIP and clinical outcomes confirmed the relevance of clone size and mutational pattern for the cardiovascular endpoint [47].

In addition to atherosclerosis, CHIP has also been associated with other cardiovascular endpoints. In patients with degenerative aortic valve stenosis undergoing transcatheter aortic valve implantation (TAVI), the presence of CHIP-mutations in *DNMT3A* or *TET2* was independently associated with increased short-term mortality in the first 8 months (hazard ratio 3.1) [58]. In patients with chronic heart failure after successfully re-vascularized myocardial infarction, CHIP was associated with reduced long-term survival and higher re-hospitalization rates due to heart failure independent from the baseline severity [59]. Furthermore, *DNMT3A* or *TET2* mutations were associated with accelerated progression of heart failure (further reduction of left ventricular ejection fraction (LVEF), heart failure-related death or hospitalization) within a 3.5 years’ follow-up irrespective of ischemic/non-ischemic etiology [60]. Finally, CHIP was prospectively associated with a 25% increased risk of incident heart failure in the population [61]. Interestingly, mutations of *ASXL1*, *TET2*, and *JAK2*, but not *DNMT3A* were found to be a risk factor for heart failure in this study [61]. Both inherited and acquired variants of *TET2* have been found in a high frequency in patients with pulmonary arterial hypertension suggesting a potential role of CHIP in disease development [62]. CHIP was also associated with an increased risk of stroke (hazard ratio 1.14) in large cohorts, particularly with hemorrhagic and small vessel ischemic stroke. In this stroke study, the strongest association was observed for *TET2* [63].

Importantly, the data on the association of CHIP and cardiovascular disease do not only rely on association studies but also build on multiple lines of evidence from in vitro and in vivo models showing a mechanistic link between somatic mutations in CHIP, proinflammatory state of myeloid cells and inflammation-induced effects on atherosclerosis and cardiac remodeling [42,64,65,66,67]. The majority of these models focused on the effect of *TET2* loss or mutations while the mechanistic role of *DNMT3A*- or *ASXL1*-mutated CHIP in cardiovascular disease development is less well understood [10]. Moreover, the interaction between CHIP and cardiovascular disease is not unidirectional but potentially influenced by reciprocal pathophysiologic effects as reviewed in detail elsewhere [68]. In addition, CHIP is associated with epigenetic modifications and telomere shortening as markers of cellular aging, and the combination of CHIP and epigenetic aging may modify the risk for adverse outcomes [69]. Finally, Heyde et al. suggested that CHIP could be a sequela rather than a cause of atherosclerosis. In this study, atherosclerosis and inflammation led to higher division rates of hematopoietic stem cell in man and murine models. This accelerated cell division could promote the emergence of clones with a moderate proliferative advantage due to somatic driver mutations and thus to a detection of CHIP in the earlier lifetime [70]. The pathophysiologic effect of *JAK2* V617F on cardiovascular risk is better understood. A number of studies indicated that the mutation alters the function of leukocytes, erythrocytes, platelets, and macrophages contributing to a thrombogenic and/or atherogenic phenotype [71,72,73,74]. In addition, vascular endothelial cells carrying the *JAK2* V617F mutation have been detected in patients with MPN, and expression of the mutation in vascular endothelial cells altered their function and response to shear stress to a pro-adhesive and pro-inflammatory phenotype promoting thrombosis and platelet adhesion in the blood vessels [75]. In summary, the mechanisms linking CHIP and cardiovascular disease are still only partially understood. Most likely, inflammation promotes CHIP and vice versa resulting in a vicious cycle of cardiovascular disease and clonal evolution of the hematopoietic compartment [76].

Despite these limitations in our understanding of the underlying pathophysiology, it is noteworthy that increased levels of biomarkers of inflammation have been observed in patients. CHIP carriers showed elevated serum levels of interleukin (IL)-6 and tumor necrosis factor alpha [77]. Moreover, high-sensitivity C-reactive protein (CRP) levels were associated with CHIP [78]. An interesting observation has been described for a germline variant of the IL-6 receptor gene (*IL6R* p.Asp358Ala) that mitigates IL-6 signaling. Presence of this germline variant seemed to attenuate the cardiovascular risk associated with CHIP [79]. The link between CHIP and inflammatory cytokines is of particular interest with regard to the CANTOS trial where the monoclonal antibody canakinumab targeting IL-1β was found to significantly lower the rate of recurrent cardiovascular events in patients with previous myocardial infarction and a CRP level of ≥ 2 mg/L [80]. While the trial met its primary endpoint and established the proof-of-concept for anti-inflammatory treatment in atherosclerosis, it did not result in the clinical application of canakinumab for cardiovascular prevention, partially due to the usage of on-treatment variables (reduction of CRP concentrations < 2 mg/L) for patient stratification [81]. A recently published retrospective analysis of the CANTOS cohort found an enrichment of *TET2*-mutated CHIP, likely explained by the usage of CRP-based inflammation as an inclusion criterion. Importantly, patients with *TET2*-mutated CHIP showed the highest reduction of major adverse cardiovascular events in an exploratory analysis suggesting that CHIP patient with *TET2* mutations may respond better to canakinumab than those without CHIP [82]. While further prospective data are needed to confirm this hypothesis, the analysis at least provides the rationale for the usage of a CHIP-based inclusion criterion for future studies on anti-inflammatory drugs in patients with atherosclerosis and cardiovascular disease.

In summary, CHIP clearly represents a previously unrecognized major risk factor for atherosclerosis and other cardiovascular diseases. It may help to identify patients at high risk for cardiovascular events despite the absence of traditional risk factors [10,83]. The most relevant clinical question how to reduce the cardiovascular risk in CHIP carriers is still unanswered. Reduction of other cardiovascular risk factors—including the optimal control of smoking, hypertension, and diabetes mellitus—as well as dietary modification and regular physical exercise seems appropriate although prospective data on the effect size are lacking. In addition, it has been suggested to consider aspirin or cholesterol-lowering statins based on individual decision [24]. Novel treatments that either reduce the CHIP-mediated inflammation specifically or target the CHIP clone directly may be tested in the future.

## 4. CHIP and Other Non-Malignant Diseases

In addition to the hematologic risk and the cardiovascular risk, CHIP has been associated with immunological and autoinflammatory diseases including rheumatoid arthritis [84], systemic sclerosis [85], osteoarthritis and subsequent total hip arthroplasty [86], and systemic lupus erythematosus [87]. The effect of CHIP on immunity and risk of infections is largely unclear. Mosaic chromosomal alterations have been associated with an increased risk of diverse incident infections including sepsis, pneumonia, digestive system infections, and genitourinary infections with a total hazard ratio of 1.25 [88]. These data cannot be interpolated to CHIP in general, as mosaic chromosomal alterations represent a distinct subset of clonal hematopoiesis only partly overlapping with CHIP as defined here [11,89]. High rates of CHIP carriers have been described in patients with HIV [90], and a study suggested that CHIP could impair the effect of antiretroviral therapy [91]. The results of CHIP and SARS-CoV-2 infections are controversial but indicate an association of CHIP with severe COVID-19 in larger cohorts [92,93,94]. In summary, further data are needed to understand if and how the presence of CHIP influence the risk of bacterial and viral infections in cancer patients—in particular when taking also additional variables such as the duration of neutropenia after chemotherapy or effects of immune therapy into account.

Moreover, CHIP has recently been associated with chronic kidney disease (CKD) Population data of the UK biobank indicated a negative association with glomerular filtration rate estimated from cystatin-C. CHIP also increased the risk of adverse outcomes in CKD [95]. Detailed follow-up analysis of patients with kidney failure and CHIP showed a 2.2-fold increased risk of kidney failure, a higher baseline kidney failure risk score, and a higher likelihood to develop complications of CKD (including anemia) [96]. Finally, Miller et al. studied the effect of CHIP on lung disease and found an association with the development and severity of chronic obstructive pulmonary disease (COPD) independent of age and cumulative smoke exposure [97]. A mouse model showing that the inactivation of *Tet2* in hematopoietic cells exacerbated emphysema development and inflammation indicated a mechanistic link of this association [97]. In short, CHIP has recently been implicated as risk factor in highly prevalent non-hematologic diseases. Whether CHIP affects organ function in cancer patients undergoing e.g., nephrotoxic therapy remains currently speculative.

## 5. Conclusions

Currently, CHIP is more often an incidental finding of genetic testing than the result of a direct diagnostic request. NGS analysis of cancer patients can lead to the discovery of CHIP when peripheral blood is used as a germline control for the tumor tissue. In addition, liquid profiling may identify CHIP mutations—either in the cfDNA fraction with the potential for a false positive result or on purpose when additional cell-based sequencing strategies are applied to correct for CHIP. Thus, the management of CHIP carriers is an emerging topic in the field of personalized medicine [98]. It includes a thorough risk assessment for both the hematologic and the non-hematologic part of the potential sequelae [53]. Of the plethora of non-hematologic diseases associated with CHIP, cardiovascular diseases are best understood. Still, evidence-based guidelines for the management of CHIP are currently not available [56]. The risk stratification in patients with CHIP includes the genetic pattern, blood count alterations, and assessment of cardiovascular risk factors. Recommendations for CHIP carriers with an increased risk for the development of hematologic neoplasm are briefly summarized above and have recently been reviewed in more detail [25]. The cardiovascular recommendations primarily focus on a strict implementation of primary and secondary cardiovascular prevention according to current guidelines [99]. In addition, comorbidities, life expectancy, and the wishes of the patient need to be considered for individualized care [100]. Patients with CHIP should ideally be treated in an interdisciplinary setting, involving specialists from hematology/oncology, cardiology, internal medicine, clinical pathology/laboratory medicine, clinical genetics, and bioinformatics—possibly in the context of specialized CHIP clinics or other collaborative approaches [99]. In cancer patients, the management of CHIP additionally includes oncologic considerations such as the tumor stage, the necessity of chemotherapy and/or radiation, and the risk-benefit ratio with regard to tMN development. However, further clinical research is required to move from descriptive association studies and individualized counselling towards evidence-based precision medicine [98].

Ultimately, CHIP is a potential incidental finding in liquid profiling of cancer patients with clinically relevant consequences. Strategies for communication on CHIP and how to deal with it should be in place when using a molecular assay with a high likelihood to diagnose CHIP incidentally [101].

## Figures and Tables

**Table 1:** Genes recurrently mutated in CHIP [12,22].

*ASXL1*	*CTCF*	*JAK3*	*PPM1D*	*SMC3*
*BAX*	*CUX1*	*KDM6A*	*PRPF40B*	*SRSF2*
*BCOR*	*DNMT3A*	*KIT*	*PTEN*	*STAG2*
*BCORL1*	*ETV6*	*KMT2A*	*PTPN11*	*STAT3*
*BRAF*	*EZH2*	*KRAS*	*RAD21*	*TET2*
*BRCC3*	*GATA2*	*MPL*	*RUNX1*	*TP53*
*CALR*	*GNAS*	*MYD88*	*SEPBP1*	*U2AF1*
*CBL*	*GNB1*	*NOTCH1*	*SF1*	*U2AF2*
*CEBPA*	*IDH1*	*NRAS*	*SF3A1*	*WT1*
*CREBBP*	*IDH2*	*PHF6*	*SF3B1*	*ZRSR2*
*CSF1R*	*JAK2*	*PIGA*	*SMC1A*	

**Table 2:** Characteristics of CHIP and myeloid neoplasms.

Finding	CHIP [11,12]	CCUS [11,12]	MDS [28]	MPN [12]
Clonality	+	+	+	+
Blood count ↓ ^1^	−	+	+	−/+ ^4^
Dysplasia ^2^	−	−	+	−
Blood count ↑ ^3^	−	−	−	+

^1^ Anemia, neutropenia and/or thrombocytopenia; ^2^ morphologic feature in bone marrow assessment (including also increased blast cell count or MDS-defining cytogenetic aberrations); ^3^ polycythemia, granulocytosis and/or thrombocythemia; ^4^ cyopenias are typically absent in MPN patients with essential thrombocythemia and polycythemia vera but are characteristic for primary myelofibrosis. Abbreviations: ↓, decrease; ↑, increase; CHIP, Clonal Hematopoiesis of Indeterminate Potential; CCUS, Clonal Cytopenia of Unknown Significance; MDS, Myelodysplastic Syndrome; MPN, Myeloproliferative Neoplasm.

## Data Availability

Not applicable.
